# Effects of fasudil on blood–brain barrier integrity

**DOI:** 10.1186/s12987-022-00336-w

**Published:** 2022-06-03

**Authors:** Kei Sato, Shinsuke Nakagawa, Yoichi Morofuji, Yuki Matsunaga, Takashi Fujimoto, Daisuke Watanabe, Tsuyoshi Izumo, Masami Niwa, Fruzsina R. Walter, Judit P. Vigh, Ana Raquel Santa-Maria, Maria A. Deli, Takayuki Matsuo

**Affiliations:** 1 Department of Neurosurgery, Graduate School of Biomedical Sciences, Nagasaki University, 1-7-1 Sakamoto, Nagasaki, 852-8501 Japan; 2 Department of Pharmaceutical Care and Health Sciences, Faculty of Pharmaceutical Sciences, Fukuoka University, 8-19-1 Nanakuma, Jonan-ku, Fukuoka, 814-0180 Japan; 3 BBB Laboratory, PharmaCo-Cell Company Ltd, Nagasaki, 852-8135 Japan; 4 Biological Barriers Research Group, Institute of Biophysics, Biological Research Centre, Szeged, 6726 Hungary; 5 Present Address: Wyss Institute for Biologically Inspired Engineering at Harvard University, 3 Blackfan Circle, Boston, MA 02115 USA

**Keywords:** Acute ischemic stroke, Astrocytes, Blood–brain barrier, Fasudil, Oxygen–glucose deprivation-reoxygenation, Pericytes, Rho-kinase inhibitor, Tight junction, Thromboxane A2

## Abstract

**Background:**

Cerebral infarction accounts for 85% of all stroke cases. Even in an era of rapid and effective recanalization using an intravascular approach, the majority of patients have poor functional outcomes. Thus, there is an urgent need for the development of therapeutic agents to treat acute ischemic stroke. We evaluated the effect of fasudil, a Rho kinase inhibitor, on blood brain barrier (BBB) functions under normoxia or oxygen–glucose deprivation (OGD) conditions using a primary cell-based in vitro BBB model.

**Methods:**

BBB models from rat primary cultures (brain capillary endothelial cells, astrocytes, and pericytes) were subjected to either normoxia or 6 h OGD/24 h reoxygenation. To assess the effects of fasudil on BBB functions, we evaluated real time impedance, transendothelial electrical resistance (TEER), sodium fluorescein permeability, and tight junction protein expression using western blotting. Lastly, to understand the observed protective mechanism on BBB functions by fasudil we examined the role of cyclooxygenase-2 and thromboxane A2 receptor agonist U-46619 in BBB-forming cells.

**Results:**

We found that treatment with 0.3–30 µM of fasudil increased cellular impedance. Fasudil enhanced barrier properties in a concentration-dependent manner, as measured by an increased (TEER) and decreased permeability. Fasudil also increased the expression of tight junction protein claudin-5. Reductions in TEER and increased permeability were observed after OGD/reoxygenation exposure in mono- and co-culture models. The improvement in BBB integrity by fasudil was confirmed in both of the models, but was significantly higher in the co-culture than in the monoculture model. Treatment with U-46619 did not show significant changes in TEER in the monoculture model, whereas it showed a significant reduction in TEER in the co-culture model. Fasudil significantly improved the U-46619-induced TEER reduction in the co-culture models. Pericytes and astrocytes have opposite effects on endothelial cells and may contribute to endothelial injury in hyperacute ischemic stroke. Overall, fasudil protects the integrity of BBB both by a direct protective effect on endothelial cells and by a pathway mediated via pericytes and astrocytes.

**Conclusions:**

Our findings suggest that fasudil is a BBB-protective agent against acute ischemic stroke.

**Supplementary Information:**

The online version contains supplementary material available at 10.1186/s12987-022-00336-w.

## Introduction

Cerebral infarction accounts for 85% of all stroke cases. Stroke is the second most common cause of mortality globally and a major cause of bedridden states in developed countries. Thus, the need for development of therapeutic agents for stroke is urgent. The therapy for acute ischemic stroke has now entered the era of rapid and highly effective reperfusion using endovascular technique. However, despite these developments, the percentage of patients with good functional outcome varies between 33 and 71% [1–5]. It is important to close the gap between high recanalization rates and poor functional outcomes.

Recently, blood–brain barrier (BBB) dysfunction due to cerebral infarction is gaining attention. Brain capillary endothelial cells form the BBB, which together with neighboring cell types, astroglia, pericytes and neurons build the neurovascular unit. The BBB protects the brain from circulating neurotoxins and inflammatory factors while maintaining appropriate concentrations of nutrients and ions in the brain for normal brain function [6, 7]. Brain endothelial cells are connected by tight junctions (TJs) and adherens junctions. One of the components of the TJs, claudins have been found to be important adhesion molecules [8, 9]. High claudin-5 expression in brain endothelial cells has been associated with normal maintenance of BBB integrity [10]. Occludin, another integral transmembrane element of TJs belonging to the Marvel-proteins [11], also contributes to the barrier integrity of cerebral endothelium [7]. Among the other cell types in the BBB, pericyte, an important cellular component of capillaries and microvessels, is closely associated with endothelial cells sharing a common basement membrane [12]. Pericytes have at least two functions: regulate BBB-specific gene expression patterns in endothelial cells and induce polarization of the astrocyte foot process surrounding central nervous system blood vessels [13]. Moreover, astrocytes around the blood vessels may regulate cellular physiology in conjunction with endothelial cells through intricate cell-to-cell communication and also by improving BBB integrity [14]. Therefore, cell–cell crosstalk within the neurovascular unit is important for the formation and maintenance of the BBB.

In cerebral ischemia, increased vascular permeability due to BBB damage leads to vasogenic cerebral edema. Although the fundamental mechanisms remain unclear, ischemia–reperfusion is known to cause an overproduction of reactive oxygen species, depletion of adenosine triphosphate, increase in extracellular potassium concentration, and activation of immune cells, which result in the activation of various inflammatory pathways and cytokine production [15]. In addition, these changes lead to increased BBB permeability, which in turn may result in hemorrhagic transformation, an important poor prognosis factor [6, 15]. Therefore, it is believed that BBB protection may improve the outcome in patients with cerebral infarction.

Rho-kinase is an intracellular serine-threonine kinase that was identified as a target for the low-molecular-weight GTP-binding protein, Rho [16–18]. Previous studies have found that Rho-kinase is involved in cellular physiological functions, such as contraction, proliferation, migration, and induction of gene expression [19]. Furthermore, Rho-kinase has been a target of drug discovery for rare motor neuron diseases, pulmonary hypertension, coronary artery disease, and obesity, among others [19]. Regarding the association between cerebral ischemia and Rho-kinase inhibitors, previous clinical trials have reported improved neurological and clinical outcomes of Rho-kinase inhibitor (fasudil) treatment in acute cerebral infarction [20]. Preclinical studies demonstrated that Rho-kinase activation in endothelial cells contributed to barrier dysfunction, ischemic expansion and edematous enlargement during cerebral ischemia [21, 22]. In addition, the neuroprotective effects of the fasudil in acute cerebral ischemia have been reported [23–25]. The vascular endothelial protective effect of Rho-kinase inhibitors has recently attracted attention. Although two studies described the effect of fasudil on the integrity of BBB [26, 27], there is no study using primary culture based BBB model necessary for the evaluation of in vitro BBB functions, and the detailed mechanisms have not been elucidated. Therefore, the aim of the present study was to examine the protective effects of fasudil on BBB under ischemic conditions. To test our hypothesis, we used rat primary cell-based in vitro BBB models composed of BBB-forming cells, including brain capillary endothelial cells, pericytes, and astrocytes. We evaluated the effect of fasudil on BBB functions under normoxia or oxygen–glucose deprivation (OGD) conditions using these models.

## Methods

### Animals

Wistar rats were obtained from CLEA Japan. Rats were housed under specific pathogen-free conditions in an air-conditioned room and fed standard laboratory chow ad libitum, in accordance with institutional guidelines. Based on the Guide for the Care and Use of Laboratory Animals from the Ministry of Education, Culture, Sports, Science, and Technology, Japan, all experimental procedures were reviewed by the Institutional Animal Care and Use Committee of Nagasaki University and finally approved by the University’s president.

### Cell culture and construction of BBB model

Rat brain endothelial cells (RBEC), astrocytes and pericytes were isolated from Wistar rats, as previously described [28, 29]. Pericytes (2.0 × 10^4^ cells/cm^2^) were seeded on the bottom side of the collagen-coated culture insert (Millicell, 0.4 µm pore size; Millipore) to construct an in vitro co-culture model. Astrocytes (1.0 × 10^5^ cell/cm^2^) were seeded on the collagen-coated well of a 24-well culture plate. Cells were left to adhere overnight, and endothelial cells (2.0 × 10^5^ cells/cm^2^) were seeded to the inside of the inserts placed in the well of the 24-well culture plates. BBB models were maintained in DMEM/F12 supplemented with 10% fetal bovine plasma derived from serum (PDS) (Animal Technologies, Inc., Tyler, TX), basic fibroblast growth factor (bFGF, 1.5 ng/mL; Roche Applied Sciences), heparin (100 μg/mL), insulin (5 μg/mL), transferrin (5 μg/mL), sodium selenite (5 ng/mL) (insulin–transferrin–sodium selenite media supplement), gentamycin (50 μg/mL), and 500 nM hydrocortisone (RBEC medium). Under these conditions, in vitro BBB models were established within 3–4 days after the seeding of the cells.

### Treatment

Fasudil hydrochloride was purchased from FUJIFILM Wako Pure Chemical (Osaka, Japan). U46619, a thromboxane A2 (TXA2) receptor agonist, was purchased from Santa Cruz Biotechnology (Texas, USA). These compounds were administered into both luminal (upper) and abluminal side (lower compartment) of the cell culture inserts.

### In vitro* oxygen–glucose deprivation and reoxygenation (OGD/R) studies on *in vitro* BBB model*

OGD/R experiments were performed as previously described with minor modification [27]. For 6 h OGD/24 h reoxygenation experiments, serum-free and glucose-free DMEM/F12 medium (Nakalai Tesque, Japan) which was bubbled with N2 gas was added to the BBB model. Oxygen deprivation was generated using Anaero Pack Kenki 5%^®^ (Mitsubishi Gas Chemical Co., Inc., Tokyo, Japan). After 6 h OGD reoxygenation was initiated by changing the medium on the cells to RBEC medium. For normoxic condition, a similar procedure for OGD condition was performed except using serum-free and glucose-containing (17.5 mM) DMEM/F12. Fasudil was added to the luminal and abluminal compartment of the BBB model at the start of reoxygenation. After 24 h reoxygenation experiments to test barrier integrity were performed.

### Impedance measurement

Kinetics of the viability of brain endothelial cells after fasudil treatment was monitored by real-time impedance measurement (RTCA-SP, Agilent, Santa Clara, CA, USA). Impedance measurement correlates linearly with cell number, adherence, growth, and viability [30]. The RTCA-SP system (Agilent, Santa Clara, CA, USA) registers the impedance of cells automatically every 10 min. Impedance (SI unit: Ω, ohm) is expressed as an arbitrary unit called cell index. For every time point cell index is defined as (Rn − Rb)/15, where Rn is the impedance of the wells containing cells and Rb means the background impedance of the wells containing medium but not cells. RBEC were seeded at a cell number of 5 × 10^3^/well onto a 96-well E-plate (Agilent) with golden electrodes at the bottom of the wells, and were kept in the CO_2_ incubator at 37 °C for 4–5 days. Cells were treated at the beginning of the plateau phase of cell growth with 0, 0.1, 0.3, 1, 3, 10, 30 and 100 μM concentrations of fasudil. Effects of the treatment were followed for 24 h.

### Transendothelial electrical resistance (TEER)

Transendothelial electrical resistance, which reflects the integrity of the BBB model [31], was measured using a Millicell^®^ ERS-2 Voltohmmeter (Merck Millipore, USA). The extracellular matrix-coated Millicell inserts were placed in a 24-well plate containing culture medium and were then used to measure the background resistance. The resistance measurements of blank inserts (background resistance) were subtracted from TEER values of inserts with cells. Values are given as Ω × cm^2^, and data indicate the change in TEER before and after treatment of OGD/R or compounds.

### Transendothelial permeability

The permeability of sodium fluorescein (Na-F) across the endothelial monolayer was determined. Cell culture inserts were transferred to 24-well plates containing 0.9 mL assay buffer (Dulbecco's phosphate-buffered saline (D-PBS) with calcium and magnesium supplemented with 4.5 g/L glucose and 10 mM HEPES, and adjusted to pH 7.4) in the lower compartments. In the inserts, the culture medium was replaced with 0.2 mL assay buffer containing 10 µg/mL Na-F (MW: 376 Da). Then, 15 or 45 min after addition of the tracer, the inserts were transferred to new wells containing assay buffer. The Na-F concentration was determined using a fluorescence multi-well plate reader (Ex(λ) 485 nm; Em(λ) 535 nm). Apparent permeability coefficient (P_app_) was calculated using the following equation: P_app_ = (dQ/dT)/(A × C0), where dQ/dT is the cumulative amount in the receiver compartment versus time, A is the surface of the filter, and C0 is the initial concentration of the tracer in the luminal compartments.

### Cell viability

RBECs were seeded at a density of 10,000 cells per well into 96-well plates. After exposure to OGD/R, the number of viable cells was determined using a Cell Counting Kit 8 (Dojindo Co., Kumamoto, Japan) according to the manufacturer’s instructions. Fasudil was added to the medium at the start of reoxygenation. The assay reagent is a tetrazolium compound (WST-8) that is reduced by live cells into a colored formazan product measured at 450 nm.

### Western blot analysis

Cultured cells were harvested by lysis in RIPA buffer for detection of the tight junction proteins. Lysates were centrifuged at 15,000×*g* for 15 min at 4 °C, supernatants were collected, and protein concentrations were determined with the BCA protein assay reagent (Pierce, Rockford, IL, USA). An equal amount of protein for each sample was separated on a 4–15% TGX (Tris–Glycine eXtended) gel (Bio-Rad, USA), transferred onto HybondTM-P (Amersham, UK), and incubated with antibodies. Anti-claudin-5, anti-occludin, and anti-ZO-1 mouse monoclonal antibodies (Invitrogen, USA) were used at dilutions of 1:5,000. Anti-VE-cadherin goat polyclonal antibody (Santa Cruz, USA) was used at a dilution of 1:2,500. Anti-β-actin mouse monoclonal antibody (Sigma, additional loading control) was used at a dilution of 1:10,000 in 3% bovine serum albumin in PBS. To visualize the immunoreactive bands, blots were incubated in SuperSignal West Femto Maximum Sensitivity Substrate (Pierce Biotechnology) and were detected using a FluorChem SP Imaging System (Alpha Innotech Corp., USA).

### Immunostaining

To stain occludin, cells were fixed with 4% paraformaldehyde for 20 min and permeabilized with 0.1% triton-X 100 for 10 min for immunofluorescence. To stain claudin-5, cells were fixed with cold methanol for 10 min at 4 ℃. After washing with PBS and blocking with 3% bovine serum albumin in PBS for 30 min, samples were incubated overnight at 4 °C with an anti-occludin antibody (1: 200) or anti-claudin-5 antibody (1:200). The source and catalogue number of antibodies is listed in Additional file 1: Table S1. After slides were washed with PBS, they were incubated with Alexa Fluor 488-conjugated secondary antibody (1: 1,000) for 60 min at 25 ℃. After washing with PBS, slides were mounted using ProLong Gold Antifade reagent containing 4′,6-diamidino-2-phenylindole (Invitrogen) to visualize the nuclei. Occludin-staining were evaluated under a Zeiss LSM 800 Confocal laser scanning microscope (Carl Zeiss AG, Oberkochen, Germany). Claudin-5-staining were evaluated under an All-in-One Fluorescence Microscope (KEYENCE, Japan).

### Quantitative real-time PCR

RBECs or pericytes were seeded into 12-well culture plates and cultured for 2 days. After 6 h OGD treatment, total RNA was isolated from RBECs and pericytes using the RNeasy mini Kit (Qiagen, Netherland). First-strand cDNA was synthesized from 500 ng of total RNA by reverse transcription (ReverTra Ace qPCR RT Master Mix; TOYOBO, Japan). Real-time PCR amplification was performed using the ABI PRISM 7900HT Sequence Detection System (Applied Biosystems). Each PCR reaction was performed by mixing 1 μL of cDNA and 5 pmol/L of each primer with the THUNDERBIRD SYBR qPCR Mix (TOYOBO). The PCR reactions were carried out at 95 °C for 1 min, followed by 40 cycles of 95 °C for 15 s, 60 °C for 15 s, and 72 °C for 45 s. The cDNA quantities were measured with critical thresholds (CTs) and normalized to Gapdh (∆CT). The ∆CT of the control was subtracted from the ∆CT of each sample to obtain ∆∆CT-values. The primers used in the analyses are as follows: sense 5ʹ-ATTACTGCTGAAGCCCACCC-3ʹ, antisense, 5ʹ-TGTGATCTGGACGTCAACACG-3ʹ for Cox-2; sense 5ʹ-ACATCAAGAAGGTGGTGAAG-3ʹ, antisense, 5ʹ- TTGGAGGCCATGTAGGCCATG -3ʹ for Gapdh.

### Statistical analysis

All data are expressed as the means ± standard error of the mean (SEM). Values were compared using analysis of variance followed by the Tukey–Kramer method. A p-value of < 0.05 was considered to be statistically significant.

## Results

### Effects of fasudil on the impedance of rat brain endothelial cells

**Fig. 1:**
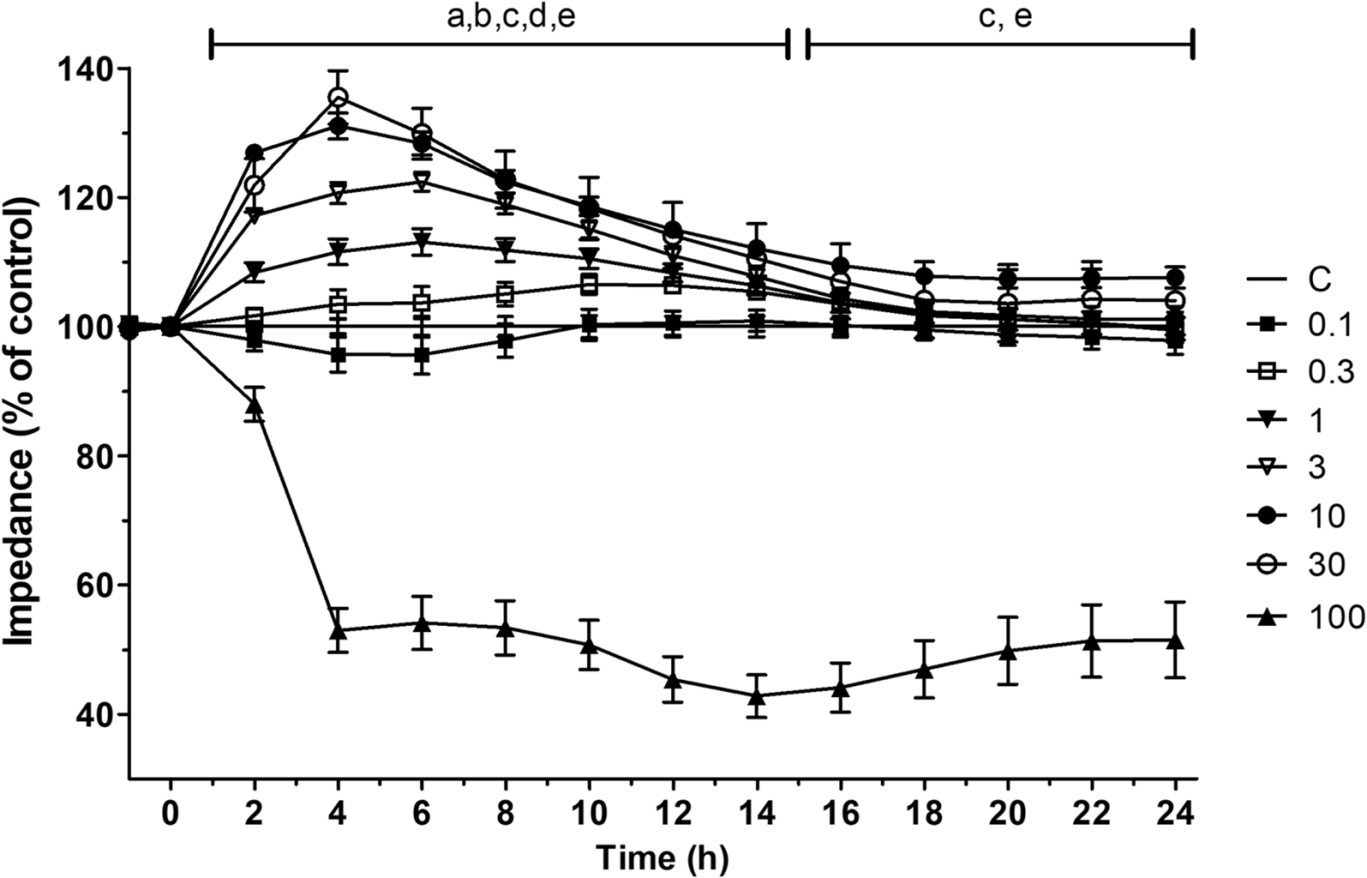
Effect of 24 h fasudil (0.1–100 µM) treatment in normoxia on the impedance of primary rat brain endothelial cells kept in monoculture on 96-well E-plate. n = 4–13. Statistical analysis: one-way Anova with Dunnett post-test. Significance is labeled with letters corresponding to the different concentrations: **a** (1 µM), **b** (3 µM), **c** (10 µM), **d** (30 µM), (100 µM), where **a**,**b**,**c**,**d**,**e**, p < 0.05. All data are presented as mean ± SEM

The effects of fasudil on the kinetics of the cellular response of RBECs were analyzed by real-time measurement of cellular impedance (Fig. 1). When RBECs are confluent, the impedance of cell layers correlates with changes in barrier integrity reflected by intercellular connections, therefore changes in impedance can imply several cellular events, including alterations in cell-to-cell adherence or cytotoxicity of treatments [30]. The impedance of the RBEC monocultures was measured continuously for 24 h after treatment with fasudil (0.1–100 μM) under normoxic conditions. Fasudil (1–30 μM) treated RBEC cultures showed significantly higher impedance than the control group indicating an improved BBB function. The cellular impedance of the cultures started increasing right after treatment with fasudil (1–30 μM), peaked at 4 h and remained significantly higher than that of the control group until 14 h. The only exception was the 10 μM treatment concentration, which showed a consistent, significantly elevated impedance compared to the control until 24 h. The 10 μM and 30 μM fasudil groups had the highest cellular impedance between 2 and 6 h after administration. In the 16–24 h interval the highest cellular impedance was measured in the case of 10 μM fasudil concentration. The highest, 100 μM fasudil concentration showed significantly lower cellular impedance than the control group (Fig. 1). The most effective concentration of fasudil was 10 μM, where the cellular impedance peaked at 4 h after treatment, and the effect persisted for 24 h. The metabolic activity of primary brain endothelial cells in confluent cultures was not decreased significantly by 24 h fasudil treatment in the concentration range of 1–100 µM measured by MTT assay (Additional file 1: Figure S1) indicating no toxic effect even for the highest, suprafarmacological concentration of 100 µM.

### Effects of fasudil on barrier functions in a co-culture BBB model

**Fig. 2:**
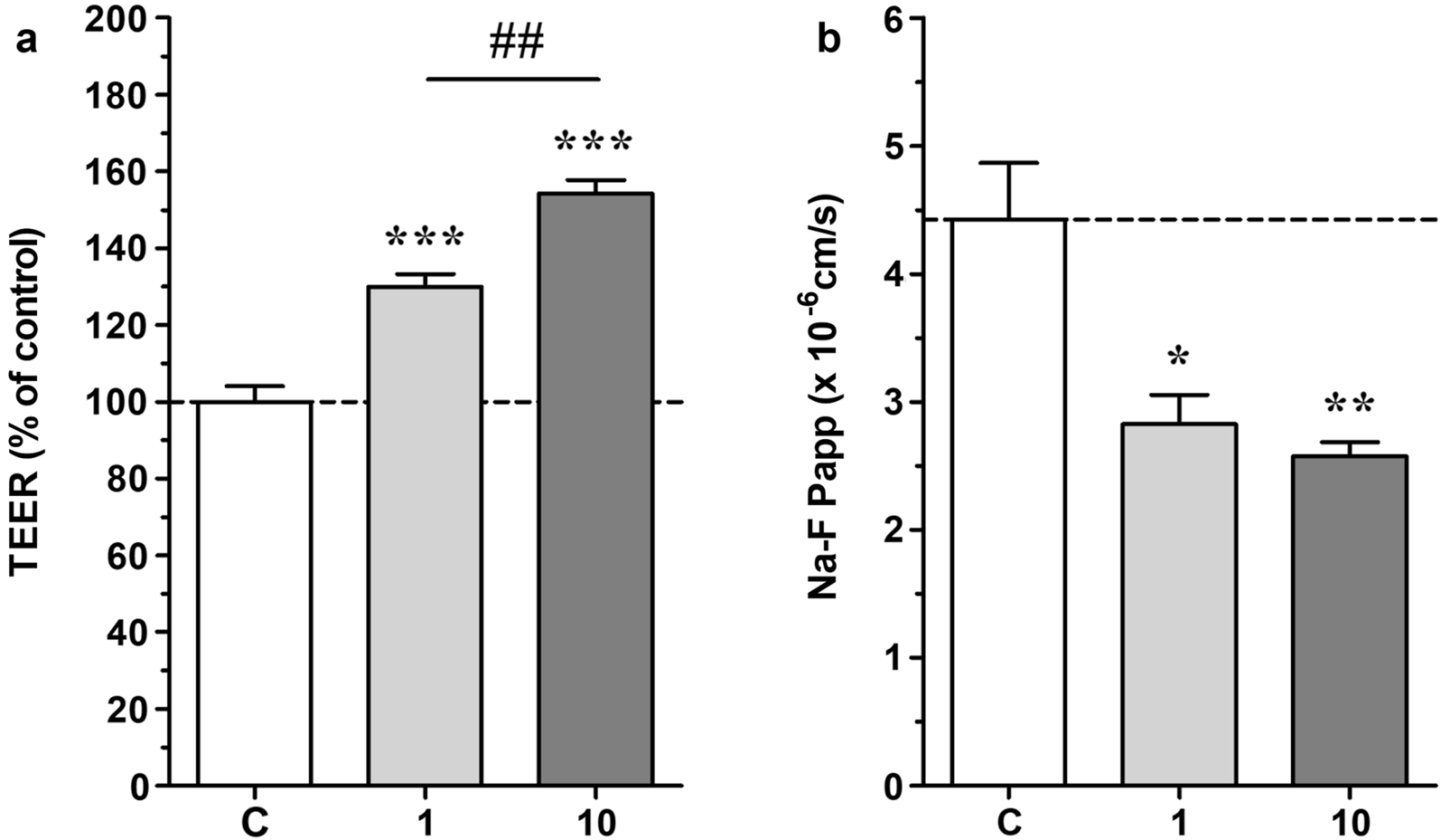
Effect of 24 h fasudil (1 and 10 µM) treatment in normoxia on the barrier integrity of primary rat brain endothelial cells kept in a triple co-culture model with primary brain pericytes and astrocytes. **A** Transendothelial electrical resistance (TEER) measurement values after treatment. n = 4, One-way Anova with Bonfferroni post-test, ***p < 0.001; ##p < 0.01. **B** Sodium fluorescein (Na-F) permeability after fasudil treatment n = 4, one-way Anova with Bonfferroni post-test, *p < 0.05; **p < 0.001. All data are presented as mean ± SEM

Next, we examined the effects of fasudil on a co-culture model, where we evaluated TEER and NaF permeability 24 h after treatment with fasudil (1 and 10 μM) under normoxic conditions. Before experiments TEER reached 119.0 ± 9.9 Ω cm^2^ for the E00 model and 453.2 ± 12.3 Ω cm^2^ (mean ± SEM) for EPA model. TEER showed a significant concentration-dependent increase after treatment with fasudil as compared with the control group (Fig. 2a). The 10 μM fasudil group showed a significantly higher increase than the 1 μM fasudil group. Compared with the control group, both 1 μM and 10 μM fasudil groups showed a significant decrease in NaF permeability (Fig. 2b). Fasudil did not change either brain endothelial cell growth measured by impedance kinetics (Additional file 1: Figure S2) or cell shape in subconfluent cultures (Additional file 1: Figure S3–S6), therefore, fasudil (1 μM, 10 μM) improved the BBB integrity in the co-culture model most probably by acting on TJs.

**Fig. 3:**
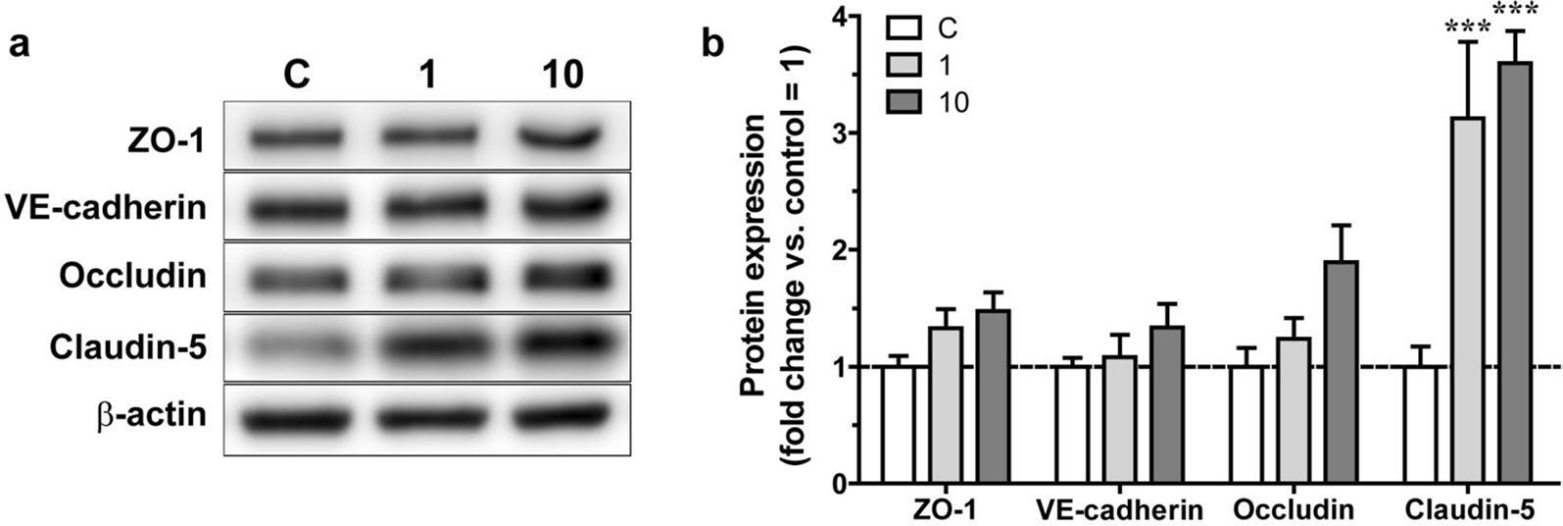
Effect of 24 h fasudil (1 and 10 µM) treatment in normoxia on junctional protein expression of primary rat brain endothelial cells kept in a triple co-culture model with primary brain pericytes and astrocytes. **A** Representative Western blot bands of chosen junctional proteins with the loading control β-actin. **B** Protein expression calculated from the intensity of the Western blot bands. ZO-1: zonula occludens-1. n = 4, Two-way Anova with Bonfferroni post-test, ***p < 0.001. All data are presented as mean ± SEM

To examine the association between the effects of fasudil on BBB integrity and the expression of TJ and adherens junction proteins was evaluated using western blotting. Compared with the control group, the 1 μM and 10 μM fasudil groups showed a significant increase in claudin-5 expression (Fig. 3a, b). No other investigated junctional protein (occludin, VE-cadeherin, ZO-1) showed an alteration.

In subconfluent and confluent mono-cultures of rat primary brain endothelial cells, the F-actin fibers form strong bundles parallel with the cell borders but are also visible within the cytoplasm (Additional file 1: Figure S6–S7). In the fasudil treatment groups at both 1 μM and 10 μM concentrations thin and continuous F-actin staining clearly delineating the cell–cell junctions was seen (Additional file 1: Figure S6–S7). These results are in concordance with the barrier tightening effects of fasudil (Figs. 2, 3). Thus, we demonstrated that fasudil improved BBB integrity by increasing the expression levels of claudin-5 and reorganizing the actin cytoskeleton.

### Effects of fasudil on BBB integrity in monoculture and co-culture models under OGD/R

**Fig. 4:**
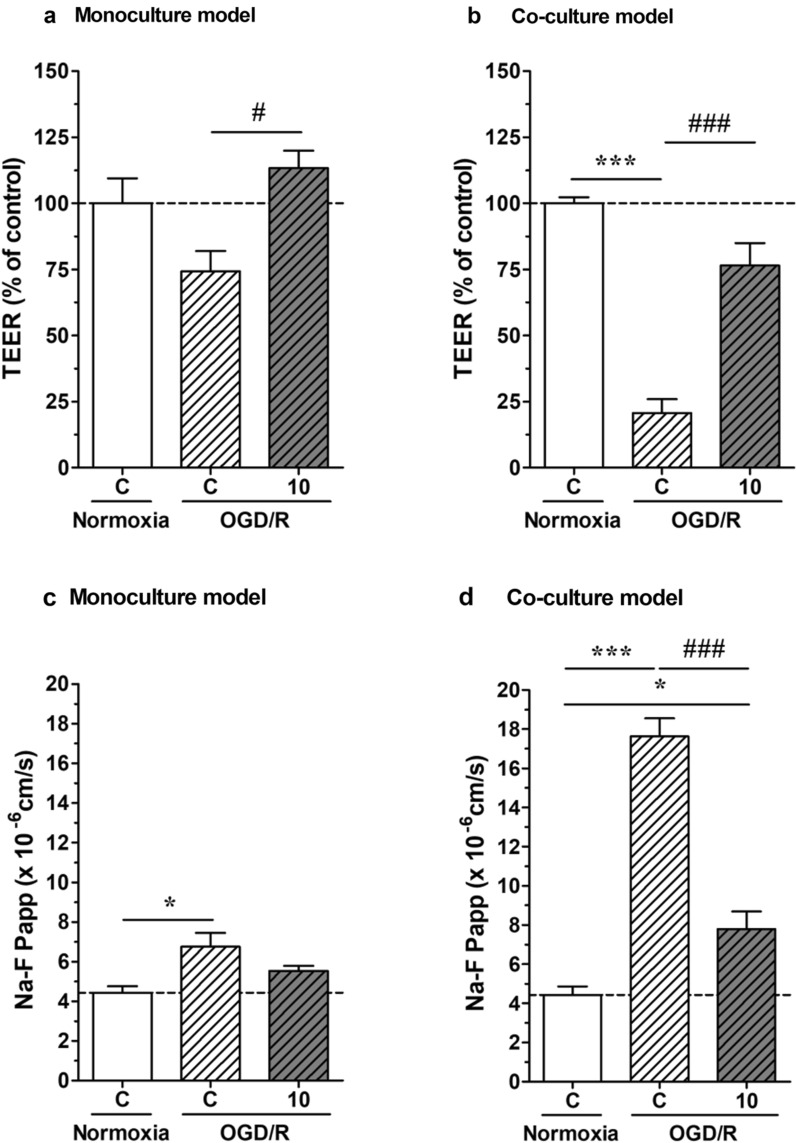
Effect of 24 h fasudil (10 µM) treatment in normoxia and oxygen–glucose deprivation/reperfusion (OGD/R) conditions on barrier properties of pimary rat brain endothelial cells kept in monoculture (**a** and **c**) or in a triple co-culture model with primary brain pericytes and astrocytes (**b** and **d**). Fasudil was given at the beginning of the 24 h reoxygenation. **A** and **B** Transendothelial electrical resistance (TEER) measurement values after treatment. **C** and **D** Sodium fluorescein (Na-F) permeability after fasudil treatment. Fasudil is protective against OGD/R conditions restoring barrier properties to the level of normoxia control almost in all conditions. n = 3–4, one-way Anova with Bonfferroni post-test, *p < 0.05; ***p < 0.001. All data are presented as mean ± SEM

We next examined the effects of fasudil (10 μM) on BBB integrity under OGD/R conditions, 6 h of OGD and 24 h of reoxygenation. OGD/R-induced BBB dysfunction was measured by TEER (Fig. 4a and b) and NaF permeability (Fig. 4c and d) in both monoculture and co-culture models. However, the barrier dysfunction in the co-culture model was higher than that in the monoculture model. Under OGD/R conditions, fasudil treatment of monoculture significantly increased TEER and tended to decrease NaF permeability compared with controls. The effect was similar but more pronounced in the co-culture model, where fasudil improved barrier dysfunction induced by OGD/R.

**Fig. 5:**
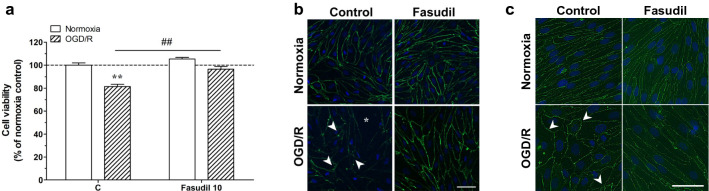
**A** Effect of 24 h fasudil (10 µM) treatment in normoxia and oxygen–glucose deprivation/reperfusion (OGD/R) conditions on cell viability performed by the cell counting kit-8 WST-8 based assay test on primary rat brain endothelial cells kept in monoculture BBB model. Unpaired t-test and two-way Anova with Bonfferroni post-test, n = 5, *p < 0.001; #p < 0.05. Data is presented as mean ± SEM. Effects of fasudil on expression of occludin (**B**) and claudin-5 (**C**) in a triple co-culture model with primary brain pericytes and astrocytes under normoxia or OGD/R condition. Immunostaining showed that the expression of occludin and claudin-5 were disrupted in RBEC in the BBB model with OGD/R compared with normoxic controls. Arrows indicate the changes in occludin and claudin-5 distribution. Moreover, fasudil improved the disruption of occludin and claudin-5 under OGD/R. Bar: 50 µm

In parallel to TEER and NaF permeability results, cell viability, detected by the cell counting kit-8 WST-8 based assay, also decreased after exposure to OGD/R and improved after treatment with fasudil (Fig. 5a). Moreover, we evaluated the fasudil effects on TJ under OGD/R conditions by immunostaining occludin and claudin-5 protein. Immunostaining showed a continuous and linear expression of occludin and claudin-5 under normoxic conditions, while the expression was disrupted under OGD/R conditions (Fig. 5b and c). In contrast, in the fasudil group the continuous and linear expression of occludin was maintained under both normoxic and OGD/R conditions (Fig. 5b and c). These results demonstrate that fasudil improves the localization of occludin and claudin-5 at the cell borders in RBECs under OGD/R conditions.

### Fasudil restored the barrier dysfunction induced by U-46619 in the co-culture model

**Fig. 6:**
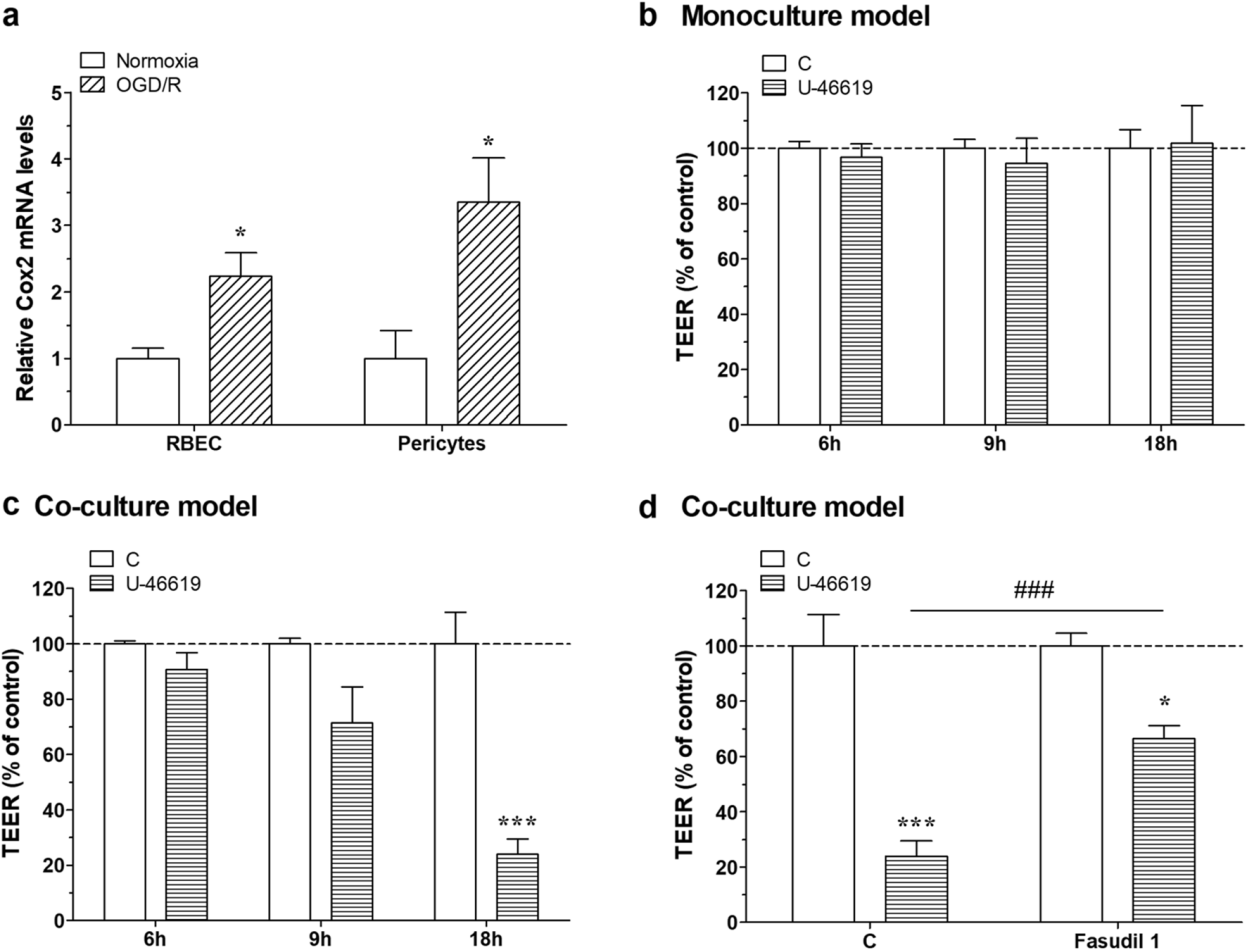
**A** After 6 h OGD treatment, pericytes significantly increased COX2 expression. **B** and **C** A monolayer or co-cultured BBB model was treated with thromboxane A2 receptor (TP) agonist (U-46619, 20 μM) for 18 h. Barrier function was investigated by TEER. There was no significant difference between control and U-46619 in monolayer model. In contrast, U-46619 decreased the TEER in co-cultured BBB model. **D** Fasudil (1 μM) improved the barrier dysfunction induced by U-46619. **a**, **b**, **c**, **d** two-way Anova with Bonfferroni post-test, n = 3, *p < 0.05; ***p < 0.001. **d** Unpaired t-test, n = 3, ##p < 0.01. All data were presented as mean ± SEM

Neuroinflammation is one of the major pathological phenomena in ischemic stroke. Brain ischemia stimulates the production of various inflammatory factors from the cells of the neurovascular unit. COX-2 plays an important role in inflammation via the production of several inflammatory factors. First, we investigated the expression levels of COX-2 in brain endothelial cells and pericytes during OGD. After 6 h of OGD, brain pericytes showed a significant increase in COX-2 expression while in RBECs a trend, but not significant increase in COX-2 was seen. Next, we examined the effect of TXA2, a prostanoid synthesized through the COX-2 pathway, on BBB integrity. The integrity of BBB was evaluated by TEER measurement after 18 h of treatment with TXA2 agonist U-46619 (20 μM). In the monoculture model, there was no significant difference in BBB integrity between the control and U-46619 groups (Fig. 6b). In contrast, U-46619 reduced TEER over time in the co-culture model (Fig. 6c). Therefore, U-46619 reduced the BBB integrity through pericytes and astrocytes in the co-culture model. We also investigated the fasudil effect on barrier integrity using U-46619-treated co-culture model. The co-culture model was treated with either U-46619 (20 μM) or U-46619 (20 μM) and fasudil (1 μM) together for 18 h. Co-administration of fasudil significantly prevented the reduction in TEER induced by U-46619 (Fig. 6d). Moreover, we evaluated the fasudil effects on claudin-5 expression on U-46619 treatment induced changes by immunostaining. A continuous and linear expression of claudin-5 was observed in the vehicle group, while the staining was disturbed by U-46619 treatment (Additional file 1: Figure S8). In contrast, in the fasudil group the continuous and linear expression of occludin was maintained even in the presence of U-46619 treatment (Additional file 1: Figure S8). Moreover, morphology of U-46619-treated endothelial cells showed altered cell shape (more roundness) compared with other groups. These results demonstrate that fasudil improves the disruption of claudin-5 and morphological change induced by U-46619 treatment.

## Discussion

This study demonstrated the protective effects of fasudil on BBB integrity. Fasudil improved BBB integrity by increasing the expression of TJ proteins. It also prevented damage to the BBB caused by ischemia–reperfusion injury modeled in vitro. Our results suggest that the protective effects of fasudil could occur via two possible pathways; one by directly affecting the endothelial cells and the other by interacting with pericytes and astrocytes to indirectly affect endothelial cells.

Cell–cell crosstalk between cerebral microvascular endothelial cells, pericytes and astrocytes is important for the formation and maintenance of BBB [13, 14]. Pericytes and astrocytes have been reported to improve BBB properties in brain endothelial cells in a co-culture model [28], but BBB integrity and the expression of TJ proteins do not increase in extensively subcultured endothelial cells, even in co-culture with pericytes and astrocytes [29]. To reveal the role of cellular interactions we used both a co-culture BBB model of primary brain endothelial cells, pericytes, astrocytes, and a primary monoculture of RBECs. An animal study showed that fasudil has a specific effect on neurovascular injury after cerebral ischemia/reperfusion by inhibiting Rho-A protein expression and increasing the expression of growth-associated protein-43 and claudin-5 [32]. An animal and cell culture study reported that Y-27632 Rho kinase inhibitor protects the BBB from ischemia-induced damage through the regulation of endothelial cell oxidative stress and TJ [27]. However, the effects of the Rho-kinase inhibitor fasudil on barrier integrity and cell–cell crosstalk between endothelial cells, pericytes, and astrocytes has not been studied and compared before on mono- and triple co-culture models of the BBB. In addition, previous studies [27] used brain microvascular endothelial cells at passage 3–6. The number of passages has been associated with a loss of primary endothelial cell properties, especially those related to BBB integrity [29]. Therefore, our goal was to test the mechanisms underlying the BBB protective effects of fasudil in our more complex, primary cell-based model.

First, we found that treatment with fasudil in the 0.3–30 μM concentration range increased cellular impedance. The lowest concentration of fasudil showing maximal effect on impedance was 10 μM. The highest concentration (100 μM) significantly reduced the impedance. In clinical practice, the maximal plasma concentration (C_max_) of fasudil is 105 ng/mL when administered intravenously for the purpose of treating cerebral vasospasm in patients with subarachnoid hemorrhage [33], which corresponds to 0.36 μM. It is expected that the administration rate will increase during cerebral ischemia and that fasudil concentration will be even higher due to intra-arterial administration, but even considering this, 100 μM is an extremely high, suprapharmacological concentration that cannot occur in vivo. We hypothesize, the impedance decreasing effect may reflect cellular toxicity. Since cellular impedance changes reflect the adherence, morphology and interendothelial barrier tightness of the cell layers, enhanced impedance by fasudil treatment indicates the strengthening of the cell–cell connections. We also confirmed the fasudil effect on BBB integrity using a co-culture model. Fasudil concentration-dependently enhanced barrier properties, as demonstrated by increased TEER and decreased permeability for a paracellular marker. Furthermore, western blotting confirmed that fasudil increased the protein expression of claudin-5. These results showed that the protective effects of fasudil on BBB integrity in the co-culture model were due to improved TJ integrity. Previous studies have reported that Rho-kinase inhibitors prevent the deterioration of BBB integrity in cerebral ischemia in a pro-oxidant environment [29]. Anti-inflammatory effects, inhibition of oxidative stress, recovery of actin microfilaments due to normalized nitric oxide levels, and the loss of stress fibers have been reported as potential mechanisms [29]. However, to the best of our knowledge, there are no studies on the protective and maintenance effects of Rho-kinase inhibitors on BBB integrity under normal conditions. The present study showed that fasudil improved BBB integrity and increased expression of TJ proteins in culture BBB models. Fasudil may improve BBB integrity by directly affecting endothelial cells, pericytes, or astrocytes and promoting the expression of TJ proteins.

Next, we confirmed the effect of fasudil on BBB integrity under OGD/R conditions. BBB integrity was compromised after OGD/R exposure in the monoculture model: reductions in TEER and an increase in NaF permeability were observed. Fasudil treatment prevented the reduction in TEER. These results suggest that fasudil improved the barrier properties in OGD/R conditions by a direct action on endothelial cells. Compared with the monoculture model, the co-culture model showed a significantly reduced BBB integrity after OGD/R measured by TEER and permeability. The protective effect of fasudil on BBB integrity was also significantly higher in the co-culture model than in the monoculture model. These results suggest that OGD/R exposure reduces BBB integrity by acting on pericytes or astrocytes, and the protective effects of fasudil on BBB integrity are exerted both indirectly through pericytes, astrocytes, and directly through endothelial cells.

Since the properties of the BBB are enhanced and maintained by cross-talk among BBB-related cells, abnormal interaction between these cells may be associated with a deterioration of BBB integrity [34, 35]. Compared to endothelial cells, pericytes are more sensitive to ischemia. The dysfunction of pericytes after cerebral ischemia leads to the opening of the TJ, resulting in BBB damage [36, 37]. In addition, pericytes surrounding the ischemic core secrete various cytokines and neurotrophic factors, which leads to modification of the cellular functions in the neurovascular unit. The above results show two characteristics of pericytes: (i) under normal conditions, they protect the BBB [6, 28], and (ii) in hyperacute ischemic stroke, they damage the BBB through direct effects such as detachment [38]. Although the underlying mechanism remains to be fully elucidated, fasudil may protect BBB integrity by preventing pericyte dysfunction or by retaining the ability to regulate the expression of TJ proteins through pericyte-derived soluble factors.

Neuroinflammation is one of the major phenomena in the pathological process of ischemic stroke. Astrocytes and pericytes produce various soluble factors during pathological conditions. For instance, COX-2 plays an important role in inflammation, and it has been reported the expression of COX-2 in astrocytes is upregulated by ischemic stress [39]. Hence, we performed further experiments to elucidated whether COX-2 is involved in the protective effect of fasudil on BBB function under OGD/R conditions. We confirmed that OGD significantly increased the expression of COX-2 in pericytes and to a lesser degree in RBECs. Combined with the previous report, our findings suggest that the increase in COX-2 expression in ischemia is largely attributed to pericytes and astrocytes. COX-2 expression is induced by cytokines and growth factors and is involved in cell proliferation, motility, adhesion, and apoptosis suppression.

In the arachidonic acid cascade, COX enzymes produce prostaglandin H2 from arachidonic acid, which is converted to other prostaglandins, such as TXA2. Pericytes and astrocytes may increase COX-2 expression during cerebral ischemia, leading to an increase in TXA2 expression. Although it is reported that TXA2 is involved in hyperglycemia-induced BBB disruption [40], a direct effect of TXA2 on BBB has not been tested. Thus, we examined the effect of a TXA2 receptor agonist, U-46619, on our BBB models. The monoculture model treated with U-46619 did not show significant changes in TEER, whereas the co-culture model treated with U-46619 showed a significant reduction in TEER. These results clearly demonstrate that TXA2 damages BBB integrity through pericytes and/or astrocytes. In addition, fasudil significantly improved the TEER reduction and claudin-5 immunostaining disturbances induced by U-46619 in co-culture models, thereby suggesting that fasudil protects BBB integrity through pericytes and/or astrocytes.

Pericytes and astrocytes contribute to the maintenance, improvement, and recovery of BBB integrity. However, they can also have opposite effects on endothelial cells and may contribute to endothelial injury in ischemic stroke.

## Conclusion

Fasudil maintains TJ integrity and protects BBB integrity by directly protecting endothelial cells and by reducing endothelial injury elicited by pericytes and astrocytes in acute ischemic stroke. The protective effects, which are most important in treating cerebral infarction, peak within the first 8 h after ischemia, and are associated with the post-treatment outcomes. Our findings show that fasudil is a BBB-protective agent against acute cerebral infarction. We hypothesize that fasudil administration is not only safe for patients with acute ischemic stroke but also have beneficial effects by reducing BBB dysfunction.

## Supplementary Information

**Additional file1:** **Table S1.** Antibodies used for immunohistochemistry and western blot. **Figure S1.** **A.** The effect of fasudil (1, 10 and 100 µM, 24-h treatment) on the metabolic activity of primary confluent cultures of rat brain endothelial cells measured by MTT assay. Data are shown as mean ± SD; n=14-16 parallels/group; two separate experiments; statistical analysis: ANOVA and Dunnett test. ***p<0.001 compared to the control group. **B.** Phase contrast micrographs from brain endothelial cells at the end of the MTT assay before cell lysis. C: control group treated with culture medium; 1, 10, 100 and 100 µM: cells treated with 1, 10 or 100 µM fasudil. Scale bar: 100 µm. **Figure S2.** Effect of fasudil (1 and 10 µM) treatment on the cell growth of primary rat brain endothelial cells kept in monoculture on 96-well E-plate measured by impedance kinetics. All data are presented as mean ± SD, n=13-17 parallels/groups. Statistical analysis: one-way Anova with Dunnett post-test. No statistically significant change was found between the groups. **Figure S3.** Effects of fasudil treatment (1 and 10 µM, 30 min) on the cellular morphology of subconfluent mono-cultures of primary rat brain endothelial cells. Representative phase contrast images at the 0 and 30 min time points. Arrows indicate the same cells in the image pairs. Scale bar: 50 µm. **Figure S4.** Effects of fasudil treatment (1 and 10 µM, 4 hours) on the cellular morphology of subconfluent mono-cultures of primary rat brain endothelial cells. Representative phase contrast images at the 0 and 4-hour time points. The image pairs do not show the exact same fields. Scale bar: 50 µm. **Figure S5.** Effects of fasudil treatment (1 and 10 µM, 24 hours) on the cellular morphology of subconfluent mono-cultures of primary rat brain endothelial cells. Representative phase contrast images at the 0 and 24-hour time points. The image pairs do not show the exact same fields. Scale bar: 50 µm. **Figure S6.** Effects of fasudil treatment (1 and 10 µM, 4 and 24 hours) on the cellular morphology of subconfluent mono-cultures of primary rat brain endothelial cells stained with phalloidin-A488. Representative confocal microscopy images. Green: F-actin, blue: cell nuclei, scale bar: 15 µm. White arrows: thin and continuous F-actin staining at the cell-cell junctions. **Figure S7.** Effects of fasudil treatment (1 and 10 µM, 4 and 24 hours) on the cellular morphology of confluent mono-cultures of primary rat brain endothelial cells stained with phalloidin-A488. Representative confocal microscopy images. Green: F-actin, blue: cell nuclei, scale bar: 15 µm. White arrows: thin and continuous F-actin staining at the cell-cell junctions. **Figure S8.** **a.** Effect of fasudil (1 µM) on cell viability without and with U-46619 (20µM) treatment. Cell viability was measured by the cell counting kit-8 WST-based assay test on pimary rat brain endothelial cells kept in monoculture BBB model. Data is presented as mean ± SEM. **b.** Effects of fasudil (1 µM) on expression of claudin-5 in a triple co-culture model with primary brain pericytes and astrocytes without and with U-46619 (20 µM) treatment. Arrows indicate the changes in claudin-5 distribution. Bar: 50 µm.

## Data Availability

The datasets used or analysed during the current study are available from the corresponding author on reasonable request.

## Abbreviations

BBB: Blood–brain barrier
COX2: Cyclooxygenase-2
OGD: Oxygen–glucose deprivation
NaF: Sodium fluorescein
OGD/R: Oxygen–glucose deprivation/re-oxygenation
RBEC: Rat brain endothelial cell
TEER: Trans-endothelial electrical resistance
TJ: Tight junction
TXA2: Thromboxane A2
VEGF: Vascular endothelial growth factor
