# Supplementary material for: Effects of fasudil on blood–brain barrier integrity
Source: Fluids Barriers CNS. 2022 Jun 3;19:43. doi: 10.1186/s12987-022-00336-w (PMC9166508; doi:10.1186/s12987-022-00336-w)
Supplement: Supplementary file 1 — Additional file1: Table S1. Antibodies used for immunohistochemistry and western blot. Figure S1. A. The effect of fasudil (1, 10 and 100 µM, 24-h treatment) on the metabolic activity of primary confluent cultures of rat brain endothelial cells measured by MTT assay. Data are shown as mean ± SD; n=14-16 parallels/group; two separate experiments; statistical analysis: ANOVA and Dunnett test. ***p<0.001 compared to the control group. B. Phase contrast micrographs from brain endothelial cells at the end of the MTT assay before cell lysis. C: control group treated with culture medium; 1, 10, 100 and 100 µM: cells treated with 1, 10 or 100 µM fasudil. Scale bar: 100 µm. Figure S2. Effect of fasudil (1 and 10 µM) treatment on the cell growth of primary rat brain endothelial cells kept in monoculture on 96-well E-plate measured by impedance kinetics. All data are presented as mean ± SD, n=13-17 parallels/groups. Statistical analysis: one-way Anova with Dunnett post-test. No statistically significant change was found between the groups. Figure S3. Effects of fasudil treatment (1 and 10 µM, 30 min) on the cellular morphology of subconfluent mono-cultures of primary rat brain endothelial cells. Representative phase contrast images at the 0 and 30 min time points. Arrows indicate the same cells in the image pairs. Scale bar: 50 µm. Figure S4. Effects of fasudil treatment (1 and 10 µM, 4 hours) on the cellular morphology of subconfluent mono-cultures of primary rat brain endothelial cells. Representative phase contrast images at the 0 and 4-hour time points. The image pairs do not show the exact same fields. Scale bar: 50 µm. Figure S5. Effects of fasudil treatment (1 and 10 µM, 24 hours) on the cellular morphology of subconfluent mono-cultures of primary rat brain endothelial cells. Representative phase contrast images at the 0 and 24-hour time points. The image pairs do not show the exact same fields. Scale bar: 50 µm. Figure S6. Effects of fasudil treatment (1 and 10 µM, 4 a [file 12987_2022_336_MOESM1_ESM.docx]

**Additional file 1**

**Effects of fasudil on blood-brain barrier integrity**

Kei Sato, Shinsuke Nakagawa, Yoichi Morofuji, Yuki Matsunaga, Takashi Fujimoto, Daisuke Watanabe, Tsuyoshi Izumo, Masami Niwa, Fruzsina R Walter, Judit P. Vigh, Ana R. Santa-Maria, Maria A. Deli, Takayuki Matsuo

**Materials and methods**

**MTT assay**

This colorimetric cell viability assay was performed as previously described by our group (Harazin et al., 2014). The yellow 3-(4,5-dimethylthiazol-2-yl)-2,5-diphenyltetrazolium bromide (MTT) dye is converted by viable cells to purple formazan crystals. Rat brain endothelial cells were grown in 96-well plates coated with collagen type IV and fibronectin. After 24-hour treatment of confluent monolayers with fasudil (1, 10 and 100 µM), cells were incubated with 0.5 mg/ml MTT solution in cell culture medium for 3 h in CO_2_ incubator. Formazan crystals were dissolved in dimethyl sulfoxide, and dye concentration was determined by absorbance measurement at 570 nm with a multiwell plate reader (Fluostar Optima; BMG Labtechnologies, Offenburg, Germany).

**Phase contrast microscopy**

For these examinations rat brain endothelial cells were seeded at a density of 1.0 × 10^4^ cells on glass bottom petri dishes (diameter 3.5 cm; Greiner, Germany) coated with a mixture of collagen type IV (100 µg/ml) and fibronectin (25 µg/ml). One day after seeding images were taken from the subconfluent cell cultures with a phase contrast microscope (20× objective, Motic, USA). Pictures from each groups were taken at 0 min time-point (baseline). Then the cells were treated with 1 or 10 µM fasudil or cell culture medium for the control group. After 4 and 24 hours of treatment futher images were taken (5-5 fields in 2 parallel dishes in each treatment groups for each time points).

**F-actin staining**

The F-actin staining was performed as previously described (Horai et al., 2013; Yamada et al., 2014). Briefly, after the 4 and 24 hours of treatments with fasudil (0, 1, or 10 µM) the cells were washed with phosphate buffered saline (PBS) and fixed with 3% paraformaldehyde in PBS for 15 min at room temperature. Following the fixation, the cells were washed again and permeabilized with 0.5% Triton-X 100 for 10 min at room temperature. After a washing step with PBS the samples were incubated with fluorescently labelled phalloidin (CF®488A; 1:100 dilution; Biotium, USA) for 20 min at room temperature. Petri dishes were mounted with Fluoromount-G (Thermo Fisher Scientific, USA) and images (5-12 / treatment group / time point) were taken by a Leica TCS SP5 confocal laser scanning microscope (Leica Microsystems, Germany).

**Table S1.** Antibodies used for immunohistochemistry and western blot.

| **Antibody** | **Company** | **Catalogue number** |
| --- | --- | --- |
| anti-claudin-5 | Invitrogen | 35-2500 |
| anti-occludin | Invitrogen | 33-1500 |
| anti-ZO1 | Invitrogen | 33-9100 |
| anti-VE-cadherin | Santa Cruz Biotechnology | sc-6458 |
| anti-β-actin | Sigma | A5441 |
| A488 conjugated donkey anti-mouse | Invitrogen | A-21202 |
| Anti-mouse IgG, HRP-linked | Cell Signaling Technology | 7076 |
| Anti-goat IgG, HRP-linked | R&D systems | HAF109 |

**Results**

**The effect of fasudil on the metabolic activity of primary rat brain endothelial cells**

The metabolic activity of primary brain endothelial cells in confluent cultures was not decreased significantly by 24-hour fasudil treatment in the concentration range of 1-100 µM measured by MTT assay (Fig. S1A) indicating no toxic effect even for the highest, suprafarmacological concentration of 100 µM. This was confirmed by phase contrast microscopy of brain endothelial cells at the end of the MTT assay before cell lysis, which revealed a similar cell density for control, culture medium-treated and fasudil treated groups (Fig. S1B). These data suggest the effect of fasudil could be primarily mediated through TJ regulation without changing the metabolic activity of brain endothelial cells. Since none of the investigated concentration groups had an effect on brain endothelial cell viability, the 1 and 10 µM fasudil concentrations, used in the other BBB and protection experiments, were tested in our further investigations.


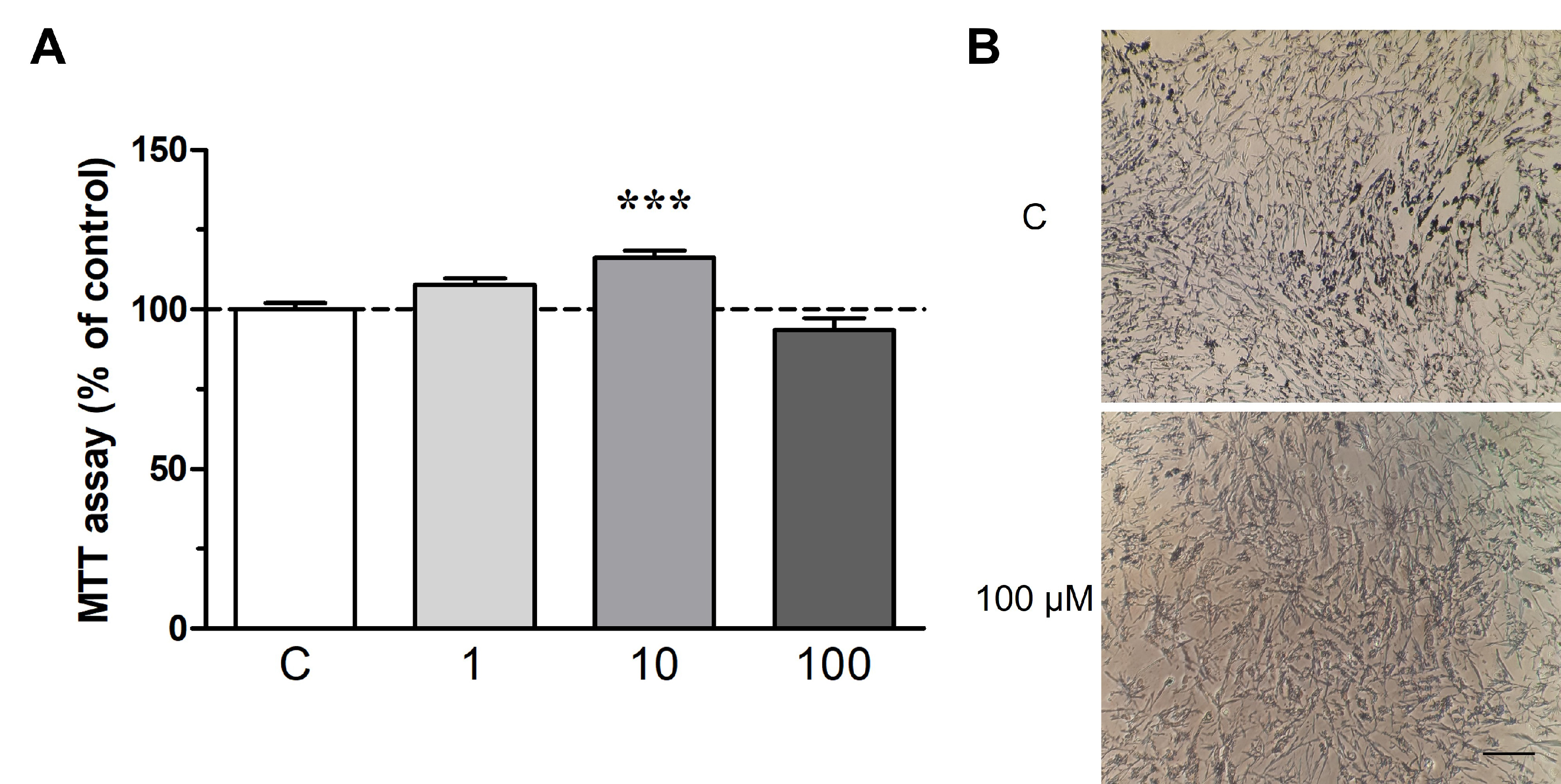


**Figure S1. A.** The effect of fasudil (1, 10 and 100 µM, 24-h treatment) on the metabolic activity of primary confluent cultures of rat brain endothelial cells measured by MTT assay. Data are shown as mean ± SD; n=14-16 parallels/group; two separate experiments; statistical analysis: ANOVA and Dunnett test. ***p<0.001 compared to the control group. **B.** Phase contrast micrographs from brain endothelial cells at the end of the MTT assay before cell lysis. C: control group treated with culture medium; 1, 10, 100 and 100 µM: cells treated with 1, 10 or 100 µM fasudil. Scale bar: 100 µm.

**The effect of fasudil on cell growth of brain endothelial cells**

To reveal the effects of fasudil on cell growth we have treated primary rat brain endothelial cells 3 hours after seeding, right after cell attachment (Fig. S2). The cell growth curves of the culture medium, 1 and 10 µM fasudil treatment groups ran parallelly until 24 hours (Fig. S2A-B) and still did not differ statistically significantly at the 48-hour time point (Fig. S2C). These data suggest that fasudil does not influence brain endothelial cell growth at these concentrations.


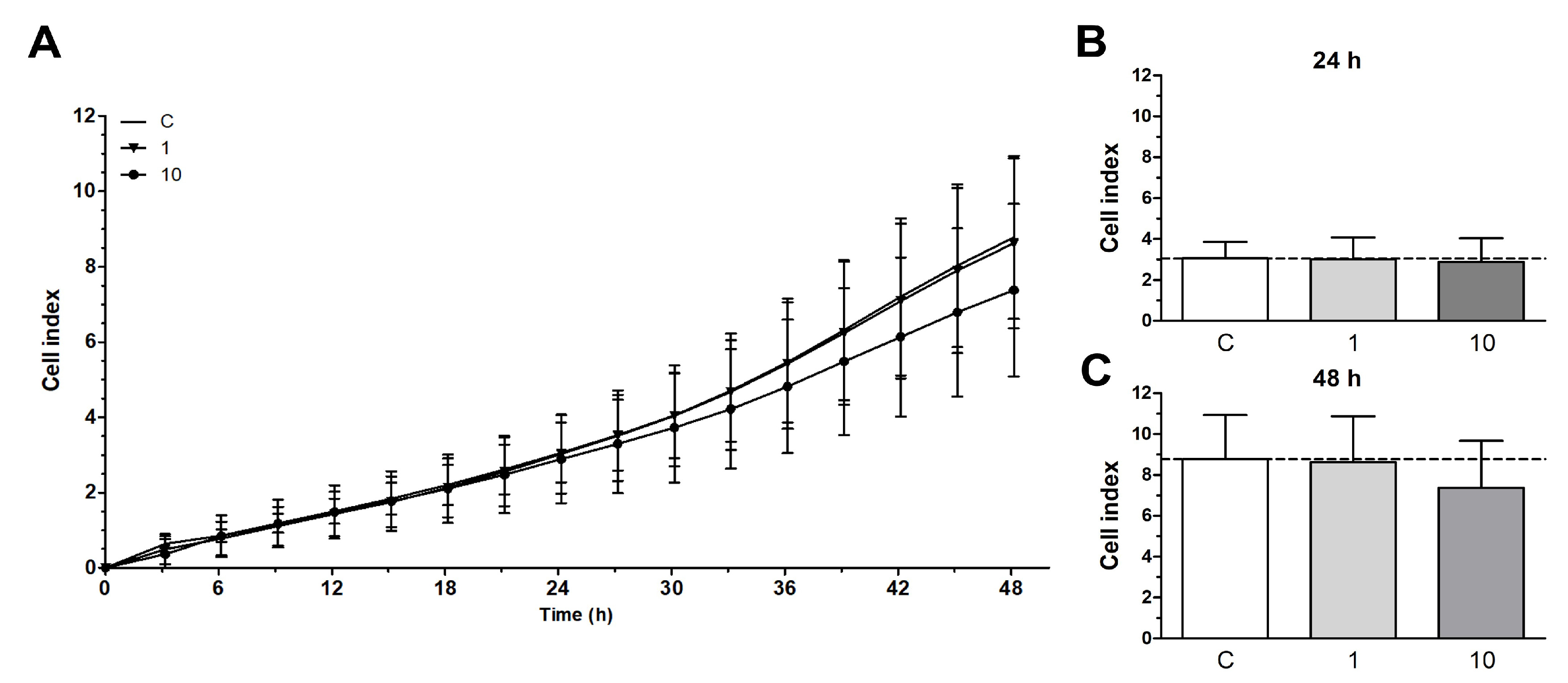


**Figure S2.** Effect of fasudil (1 and 10 µM) treatment on the cell growth of primary rat brain endothelial cells kept in monoculture on 96-well E-plate measured by impedance kinetics. All data are presented as mean ± SD, n=13-17 parallels/groups. Statistical analysis: one-way Anova with Dunnett post-test. No statistically significant change was found between the groups.

**The effects of fasudil on brain endothelial morphology in subconfluent cultures: phase contrast microscopy**

To investigate quick changes in the morphology of subconfluent cultures, rat brain endothelial cells grown on culture dishes with glass bottoms were treated with 1 and 10 µM concentrations of fasudil and monitored by phase contrast videomicroscopy until 30 min (Fig. S3). This experiment was performed at room temperature. From the 2 parallel dishes in each treatment groups 5-5 fields were marked to take images. Representative images at the 0 and 30 min time points from the same fields are shown in Fig. S3. We observed minimal cell shrinkage at 30 min as compared to 0 min in all three treatment groups (Fig. S3 arrows) which suggest that this change is most probably due to temperature change (37°C vs. 25°C) and not to the fasudil effect.


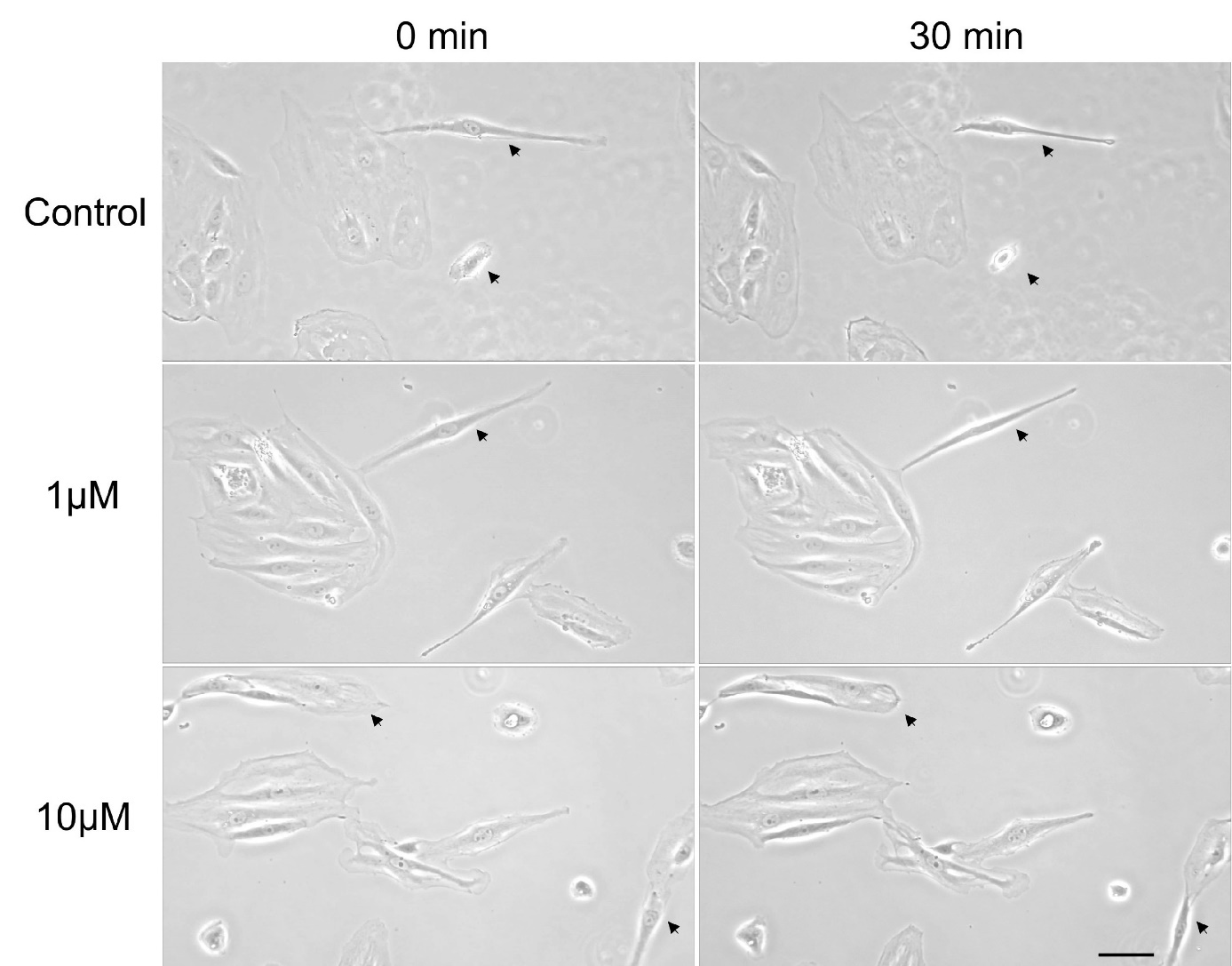


**Figure S3.** Effects of fasudil treatment (1 and 10 µM, 30 min) on the cellular morphology of subconfluent mono-cultures of primary rat brain endothelial cells. Representative phase contrast images at the 0 and 30 min time points. Arrows indicate the same cells in the image pairs. Scale bar: 50 µm.

The morphology of subconfluent cultures of rat brain endothelial cells was also tested at the 4-hour time point after treatment with 1 and 10 µM concentrations of fasudil (Fig. S4). Here the cells were kept in culture incubators at 37°C, then the images were quickly taken (5-5 fields in 2 parallel dishes in each treatment groups). Despite marking the random fields at the bottom of the dishes, unlike during the videomicroscopy (Fig. S3) the images taken do not show exactly the same groups of cells (Fig. S4). No striking difference in the morphology of the subconfluent cultures of brain endothelial cells could be observed at this time point.


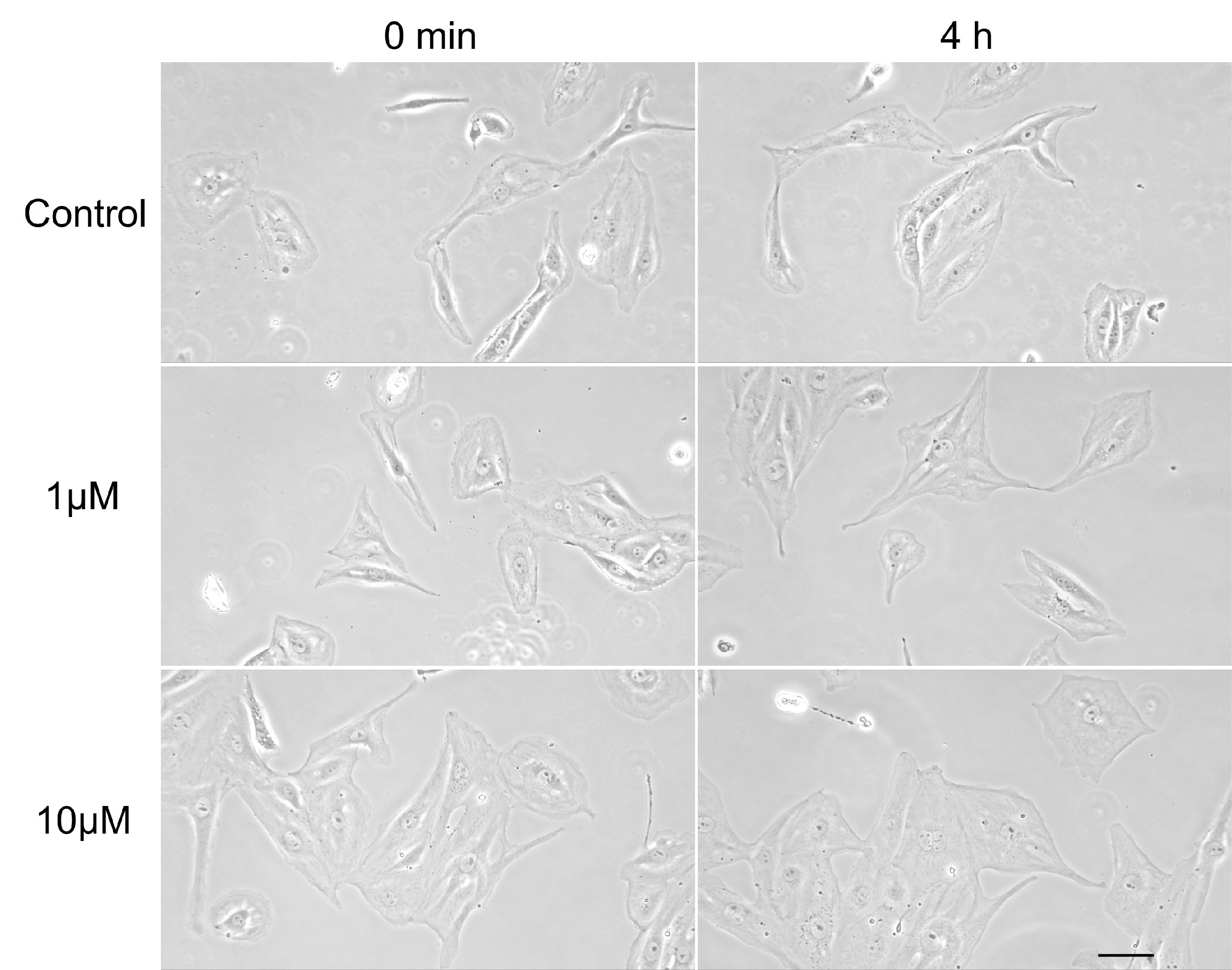


**Figure S4.** Effects of fasudil treatment (1 and 10 µM, 4 hours) on the cellular morphology of subconfluent mono-cultures of primary rat brain endothelial cells. Representative phase contrast images at the 0 and 4-hour time points. The image pairs do not show the exact same fields. Scale bar: 50 µm.

The morphology of subconfluent cultures of rat brain endothelial cells was finally tested at the 24-hour time point after treatment with 1 and 10 µM concentrations of fasudil (Fig. S5). During the treatment the cells were kept in culture incubators at 37°C, then the images were quickly taken (5-5 fields in 2 parallel dishes in each treatment groups). Despite marking the random fields at the bottom of the dishes, unlike during the videomicroscopy (Fig. S3) the images taken do not show exactly the same groups of cells (Fig. S5). Similarly to the 30-min and 4-hour time points no striking difference in the morphology of the subconfluent cultures of brain endothelial cells could be observed after 24-hour treatment with fasudil.


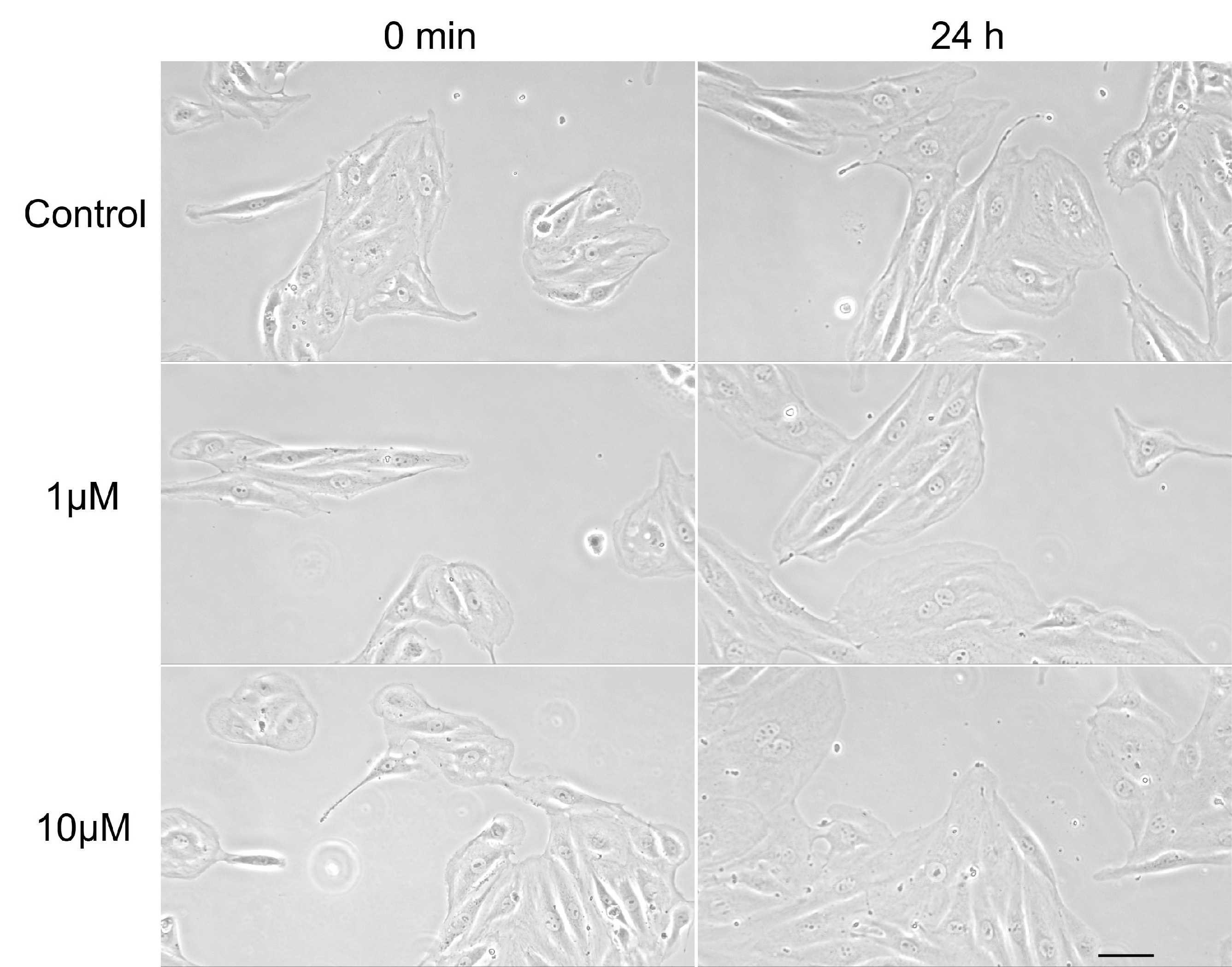


**Figure S5.** Effects of fasudil treatment (1 and 10 µM, 24 hours) on the cellular morphology of subconfluent mono-cultures of primary rat brain endothelial cells. Representative phase contrast images at the 0 and 24-hour time points. The image pairs do not show the exact same fields. Scale bar: 50 µm.

**The effects of fasudil on brain endothelial morphology in subconfluent and confluent cultures: F-actin staining**

To further investigate the junctional morphology of brain endothelial cells treated with 1 and 10 µM concentrations of fasudil, the F-actin structure was visualized by fluorescently labelled phalloidin and confocal microscopy (Fig. S6). In subconfluent cultures in the control group F-actin bundles within the cytoplasm could be observed. In the 4-hour fasudil treatment groups at both concentrations the F-actin fibers in the cytoplasm were less pronounced, while thin and continuous F-actin staining could be observed at the cell-cell junctions (Fig. S6, white arrows). These results are in good agreement with the tight junction strengthening effects of fasudil revealed by functional measurements (Fig. 2) and tight junction protein expression (Fig. 3).


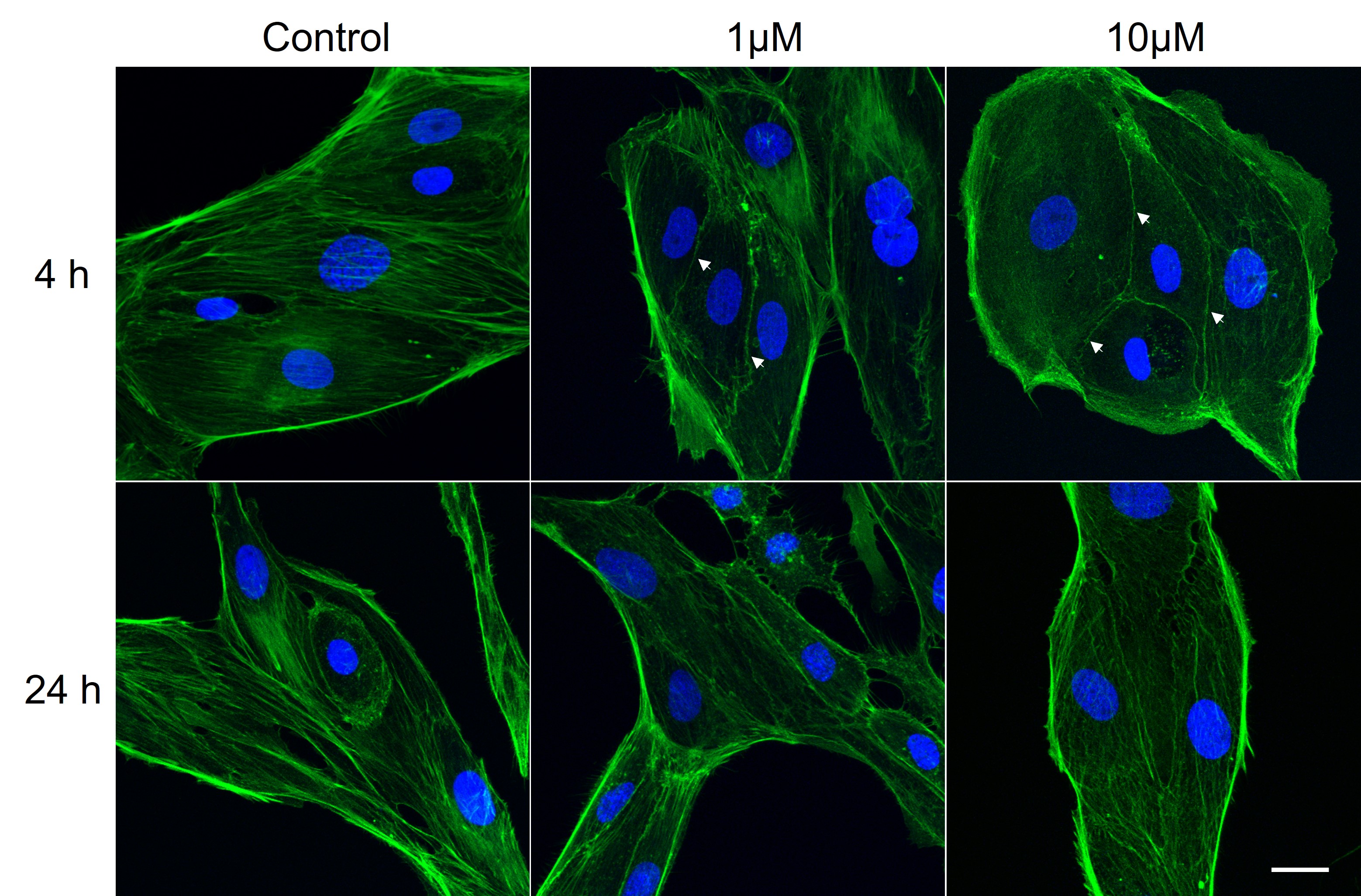


**Figure S6.** Effects of fasudil treatment (1 and 10 µM, 4 and 24 hours) on the cellular morphology of subconfluent mono-cultures of primary rat brain endothelial cells stained with phalloidin-A488. Representative confocal microscopy images. Green: F-actin, blue: cell nuclei, scale bar: 15 µm. White arrows: thin and continuous F-actin staining at the cell-cell junctions.

In confluent cultures of rat primary brain endothelial cells, the F-actin fibers form strong bundles parallel with the cell borders but are also visible within the cytoplasm (Fig. S7). In the fasudil treatment groups at both concentrations thin and continuous F-actin staining clearly delineating the cell-cell junctions was seen (Fig. S7, white arrows). These results are in concordance with the barrier tightening effects of fasudil (Fig. 2 and Fig. 3). Previous works from our groups found also similarly smooth and sharp staining pattern of F-actin at the cell border of brain endothelial cells after treatment with the clinically used phosphodiesterase inhibitor cilostazol acting through the cAMP pathway (Horai et al., 2013) or with hepatocyte growth factor (Yamada et al., 2014).

**
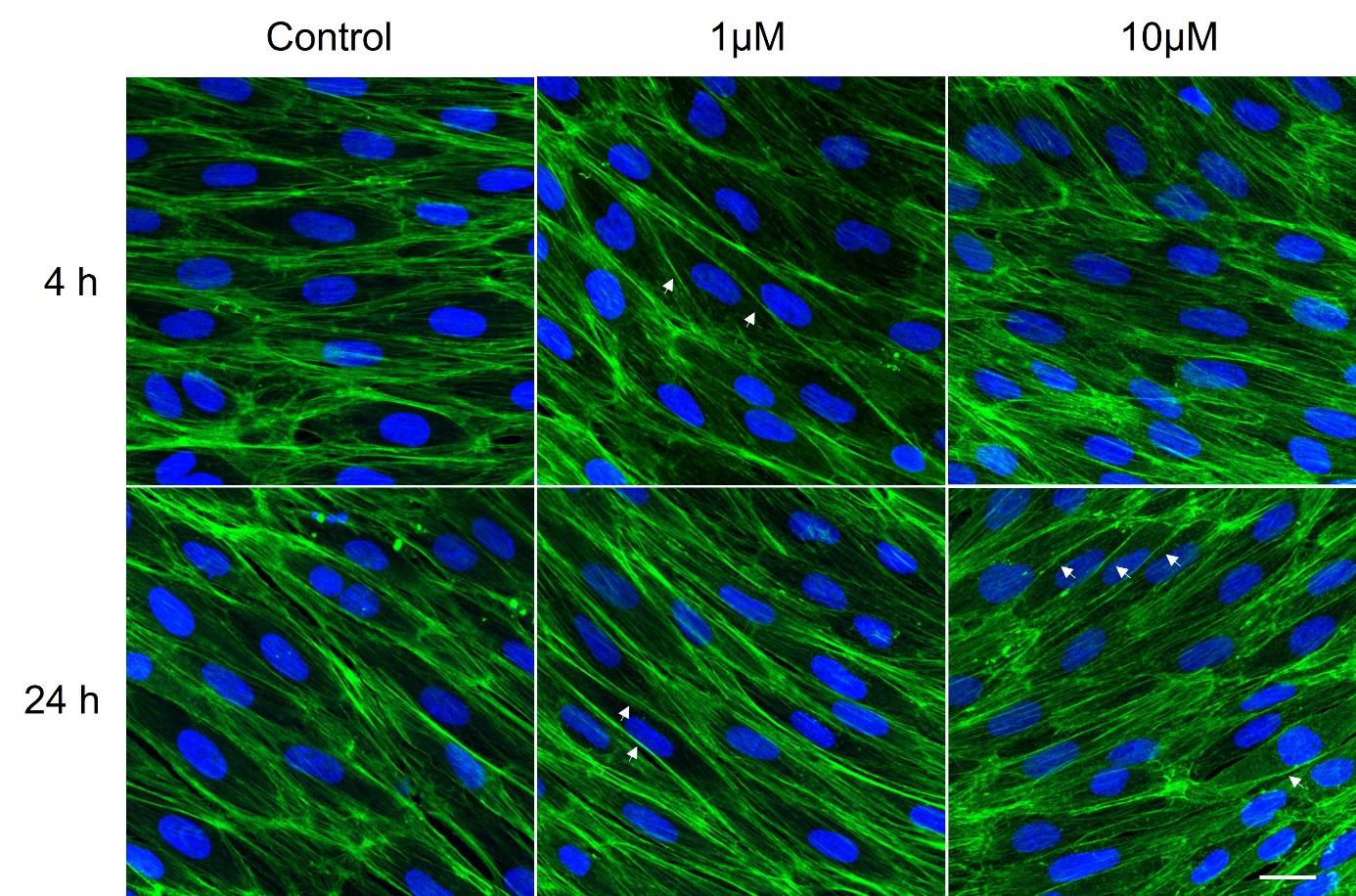
**

**Figure S7.** Effects of fasudil treatment (1 and 10 µM, 4 and 24 hours) on the cellular morphology of confluent mono-cultures of primary rat brain endothelial cells stained with phalloidin-A488. Representative confocal microscopy images. Green: F-actin, blue: cell nuclei, scale bar: 15 µm. White arrows: thin and continuous F-actin staining at the cell-cell junctions.

Rho kinase inhibitors, including fasudil, may change cell spreading and shape in subconfluent cultures as it was demonstrated on peripheral endothelium, human umbilical vein endothelial cells (Yin et al., 2007) and murine glomerular microvascular endothelial cells (Breyer et al., 2012). Interestingly, we have not found such changes in our subconfluent cultures hinting at organ-specific effect of Rho-kinase inhibitors on microvascular endothelial cells. We should note, that the primary cultures used in our studies possess blood-brain barrier phenotype (Nakagawa et al., 2009; Veszelka et al., 2018) and are able to form tight interendothelial junctions already in very small colonies (Fig. S6) that may explain the differences between the present findings and other studies.

**The effect of U-46619 on EC viability, morphology, and tight junction expression**

Both 18 hours U-46619 (20 μM) or fasudil (1 μM) treatment in RBEC monolayer did not affect EC viability estimated by cell counting kit-8 assay (Fig. S8a). On the other hand, U-46619 affected the morphology and claudin-5 expression in RBEC co-cultured with pericytes and astrocytes (Fig. S8b). Immunostaining showed that the expression of claudin-5 was disrupted in RBEC in the BBB model treated with U-46619 compared with vehicle controls. Moreover, morphology of U-46619-treated endothelial cells showed altered cell shape (more roundness) compared with vehicle controls. Fasudil improved the disruption of claudin-5 and morphological change induced by U-46619.


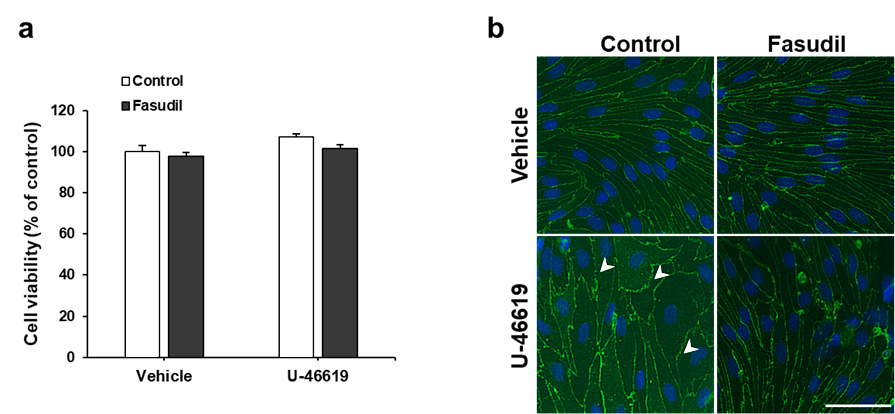


**Figure S8. a.** Effect of fasudil (1 µM) on cell viability without and with U-46619 (20µM) treatment. Cell viability was measured by the cell counting kit-8 WST-based assay test on pimary rat brain endothelial cells kept in monoculture BBB model. Data is presented as mean ± SEM. **b.** Effects of fasudil (1 µM) on expression of claudin-5 in a triple co-culture model with primary brain pericytes and astrocytes without and with U-46619 (20 µM) treatment. Arrows indicate the changes in claudin-5 distribution. Bar: 50 µm.

**References**

Breyer J, Samarin J, Rehm M, Lautscham L, Fabry B, Goppelt-Struebe M. Inhibition of Rho kinases increases directional motility of microvascular endothelial cells. Biochem Pharmacol. 2012 83(5):616-26.

Harazin A, Bocsik A, Barna L, Kincses A, Váradi J, Fenyvesi F, Tubak V, Deli MA, Vecsernyés M. Protection of cultured brain endothelial cells from cytokine-induced damage by α-melanocyte stimulating hormone. PeerJ. 2018 6:e4774.

Horai S, Nakagawa S, Tanaka K, Morofuji Y, Couraud PO, Deli MA, Ozawa M, Niwa M. Cilostazol strengthens barrier integrity in brain endothelial cells. Cell Mol Neurobiol. 2013 33(2):291-307.

Nakagawa S, Deli MA, Kawaguchi H, Shimizudani T, Shimono T, Kittel A, Tanaka K, Niwa M. A new blood-brain barrier model using primary rat brain endothelial cells, pericytes and astrocytes. Neurochem Int. 2009 54(3-4):253-63

Veszelka S, Tóth A, Walter FR, Tóth AE, Gróf I, Mészáros M, Bocsik A, Hellinger É, Vastag M, Rákhely G, Deli MA. Comparison of a Rat Primary Cell-Based Blood-Brain Barrier Model With Epithelial and Brain Endothelial Cell Lines: Gene Expression and Drug Transport. Front Mol Neurosci. 2018 11:166.

Yamada N, Nakagawa S, Horai S, Tanaka K, Deli MA, Yatsuhashi H, Niwa M. Hepatocyte growth factor enhances the barrier function in primary cultures of rat brain microvascular endothelial cells. Microvasc Res. 2014 92:41-9.

Yin L, Morishige K, Takahashi T, Hashimoto K, Ogata S, Tsutsumi S, Takata K, Ohta T, Kawagoe J, Takahashi K, Kurachi H. Fasudil inhibits vascular endothelial growth factor-induced angiogenesis in vitro and in vivo. Mol Cancer Ther. 2007 6(5):1517-25.
